# Evaluation of biomechanical properties and biocompatibility: are partially absorbable cords eligible for anterior cruciate ligament reconstruction?

**DOI:** 10.3389/fbioe.2023.1216937

**Published:** 2023-10-02

**Authors:** Fei Xu, Yanlin Li, Yang Yu, Guoliang Wang, Guofeng Cai

**Affiliations:** ^1^ Kunming Medical University, Kunming Yunnan, China; ^2^ Department of Pain Treatment, The First People’s Hospital of Yunnan Province, Kunming Yunnan, China; ^3^ Department of Sports Medicine, the First Affiliated Hospital of Kunming Medical University, Kunming Yunnan, China

**Keywords:** ACL reconstruction, augmentation technique, reinforcing cord, polydioxanone, ultra-high molecular weight polyethylene

## Abstract

**Introduction:** Independent augmentation technology based on reinforcing devices has been reported to signifi-cantly reduce the elongation behavior of graft and improve knee stability after anterior cruciate ligament reconstruction (ACLR). Using biodegradable devices could reduce the risk of severe inflammatory reactions due to particle accumulation from foreign bodies. Given the limitations of the mechanical properties of biodegradable materials, partially biodegradable composite devices may offer a compromise strategy.

**Methods:** Three types of partially absorbable core-sheath sutures, including low-absorbable cord (LA-C), medium-absorbable cord (MA-C) and high-absorbable cord (HA-C), were braided using unabsorbable ultra-high molecular weight polyethylene (UHMWPE) yarn and absorbable polydioxanone (PDO) monofil-ament bundle based on the desired configuration. The feasibility of these partially absorbable cords were verified by biomechanical testing, material degradation testing, and cell experiments, all performed *in vitro*.

**Results:** Reinforcement of an 8 mm graft with the cords decreased dynamic elongation by 24%–76%, was positively related to dynamic stiffness, and increased the failure load by 44%–105%, during which LA-C showed maximum enhancement. Human ligament-derived fibroblasts showed good proliferation and vitality on each cord over 2 weeks and aligned themselves in the direction of the fibers, especially the UHMWPE portion.

**Discussion:** This study supports the potential of partially degradable UHMWPE/PDO cords, particularly LA-C, for graft protection. Nervertheless, a higher proportion of biodegradable material results in lower stiffness, which may impair the protective and lead to mechanical instability during degradation.

## 1 Introduction

In the United States, it is reported that more than 100,000 ACLRs are performed annually (Montalvo et al., 2019), and primary grafts are autogenous hamstring tendon or bone-patellar tendon-bone complex (Ardern et al., 2011; Vaishya et al., 2015). However, in young and athletic individuals (Wiggins et al., 2016; Zbrojkiewicz et al., 2018), small diameter grafts (Magnussen et al., 2012; Mariscalco et al., 2013), graft ligamentalization (Ménétrey et al., 2008; Claes et al., 2011), accelerated rehabilitation (Beynnon et al., 2011; Culvenor et al., 2018), and accidental injury are all risk factors leading to postoperative graft relaxation or rupture. In short, the mismatch between the graft-loading capacity and load demand in the rehabilitation process leads to adverse results. Improving the mechanical properties of grafts through reinforcement technology may be the solution.

The Kennedy LAD, which appeared in 1980, was proved to significantly enhance the mechanical strength of the patellar tendon graft used for ACLR (Zbrojkiewicz et al., 2018) but was abandoned because of uncertainty regarding its clinical efficacy and complications such as joint effusion and synovitis (Dahlstedt et al., 1990; Batty et al., 2015)]. Although artificial LARS ligament has shown better efficacy and lower complications (Batty et al., 2015), it has also been questioned due to reports of inflammatory reactions from foreign bodies (Sinagra et al., 2018). Recently, reinforcement technology has received renewed attention due to the application of suture tape braided using UHMWPE fibers (Frangie et al., 2020). This high-strength belt could effectively share the graft load in dynamic stretching and play the role of a safety belt to reduce the risk of relaxation (Cook et al., 2017; Bachmaier et al., 2018; Soreide et al., 2019; Smith et al., 2020a; Noonan et al., 2020; Lai et al., 2021; Matava et al., 2021; Bachmaier et al., 2022). In clinical practice, suture tape can significantly improved knee joint function and promoted the return to regular activity after ACLR surgery (Kitchen et al., 2022; von Essen et al., 2022; Parkes et al., 2021; Bodendorfer et al., 2019).

Many studies have demonstrated the effectiveness and necessity of the reinforcement technique in ACLR (EAM et al., 2022). However, given the long-term retention of the reinforcement device in the joint, wear particles generated by non-degradable materials in the long term are potential risk factors for foreign body inflammation (Marieswaran et al., 2018; Sinagra et al., 2018). To achieve biodegradability, researchers have attempted to use materials with long degradation periods, such as polyurethane urea (Peterson et al., 2014), PDO (Buma et al., 2004), and poly-L-lactide/L-lactide-co-glycolide (Dürselen et al., 2003; Dürselen et al., 2006) to manufacture biodegradable reinforcement devices for ACLR. However, negative results and *in vivo* failure of these devices have been reported (Buma et al., 2004; Dürselen et al., 2006). Therefore, such biodegradable devices cannot be used as reinforcements for ACLR, mainly because of mechanical and structural instability caused by material degradation. In addition, no study has yet described *in vitro* biomechanical testing to confirm the exact augmentation effect of these devices on grafts.

Inspired by the strategy of composite materials in tissue engineering (Silva et al., 2020), a partially biodegradable reinforcing device may be a solution to balance biodegradability and mechanical requirements. Theoretically, the higher the degradable proportion in the device, the less amount of material is accumulated *in vivo*, while providing sufficient graft protection. In this study, UHMWPE yarn with superior mechanical strength (Savage et al., 2013) and PDO monofilament with a suitabledegradation period of 6 months (REA et al., 2018) were used to manufacture reinforcing cords with different material configurations. *In vitro* biomechanical and degradation testing and *in vitro* cell testing were performed to verify the feasibility of the partially biodegradable reinforcing cord.

## 2 Materials and methods

### 2.1 Manufacture of sutures and reinforcing cords

**FIGURE 1:**
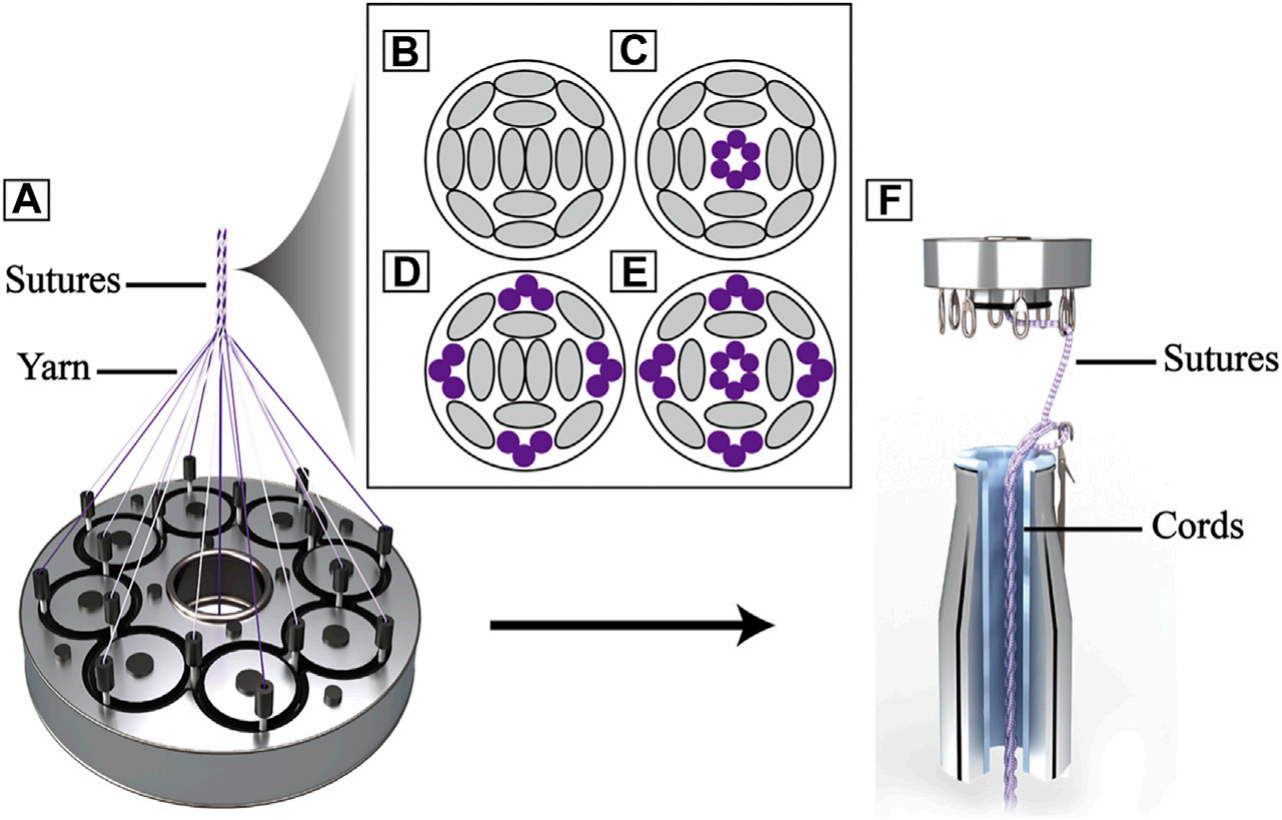
Schematic image of reinforcing cords: **(A)**. Schematic of sheath-core sutures braiding; **(B)**, **(C)**, **(D)**, **(E)** Section schematic of sutures **(B)**: UA-S, **(C)** LA-S, **(D)** MA-S, **(E)** HA-S), grey oval represent UHMWPE yarn, purple circle represent PDO monofilament; **(F)**. Schematic of reinforcing cords weaving.

The core-sheath sutures were braided out of PDO bundle (3 monofilaments, USP 7–0 monofilament, Samyong, Korea) and UHMWPE yarn (100 denier, Honeywell, New Jersey, United States ) by a custom 16 spindle braiding machine (Figure 1A), the material configurations of each suture were shown in Table 1. UA-S consisted of UHMWPE sheath and UHMWPE core (Figure 1B), LA-S consisted of UHMWPE sheath and PDO core (Figure 1C), MA-S consisted of UHMWPE/PDO sheath and UHMWPE core (Figure 1D), HA-S consisted of UHMWPE/PDO sheath and PDO core (Figure 1E). As shown in Figure 1F, each single suture was further crocheted into a chain stitch cord by a custom knitting machine. Four reinforcing cords were obtained:, namely, UA-C, LA-C, MA-C, and HA-C. Uniform manufacturing parameters were used between groups in the above two processes.

**TABLE 1:** Sheath-core material configuration of each suture.

	UA-S	LA-S	MA-S	HA-S
Sheath	UHMWPE (16)	UHMWPE (12) + PDO (4)	UHMWPE	UHMWPE (12) + PDO (4)
Core	UHMWPE (2)	PDO (2)	UHMWPE (2)	PDO (2)

() number of UHMWPE, yarn or PDO, bundle.

### 2.2 Characterization of sutures and cords

Photos of each suture and cord (n = 3) were taken using a depth-of-field microscope (Leica DVM6, Weztla, Germany). The diameter and braiding angle of the sutures and the width and pitch of the cords were measured by ImageJ software (National Institute of Health, Bethesda, MD, United States ), randomly selecting three positions of each photo for measurement and calculating the mean value. The mechanical properties of the sutures and cords (n = 3) were tested by a dynamic machine equipped with 20 kN mechanical units (Instron 8,872, Boston, United States ). Bypassing the suture or cord through the suspension shaft of the upper and lower clamp and tying four half-hitch knots, a loop of 10 cm in length was formed, which was stretched to failure at a speed of 50 mm/min. Data were recorded by Wavematrix software (Instron, Boston, United States ) with a sampling rate of 500 Hz, and stiffness was calculated in the linear region.

### 2.3 Biomechanical testing

#### 2.3.1 Graft preparation

Bovine flexor tendons from the hind limbs of adult bovine were selected as grafts for their mechanical and structural properties as they are similar to human hamstring tendons (Donahue et al., 2001). A single tendon was cut to 220 mm in length and trimmed parallel to the direction of collagen fibers until it could be folded into a tripled graft that was 70 mm in length and 8 mm in diameter. Each end of a tendon was passed through an adjustable loop (TightRope RT, Arthrex, Naples, United States ) and folded into a tripled graft. A rip-stop suture was performed with 2# Ethibond suture (Ethicon, New Brunswick, United States ) 5 mm away from both ends (Yoo et al., 2019). The graft was fixed in a preparation station and tensioned with a load of 20 N, then whip-stitched three times at the last 15 mm of both ends. Another rip-stop suture was performed 10 mm away from both ends when the load was increased to 50 N. Grafts were kept moist throughout the preparation process, stored at −20 °C after preparation, and thawed at room temperature for 2 hours before mechanical testing.

#### 2.3.2 Construct fixation and testing protocol

**FIGURE 2:**
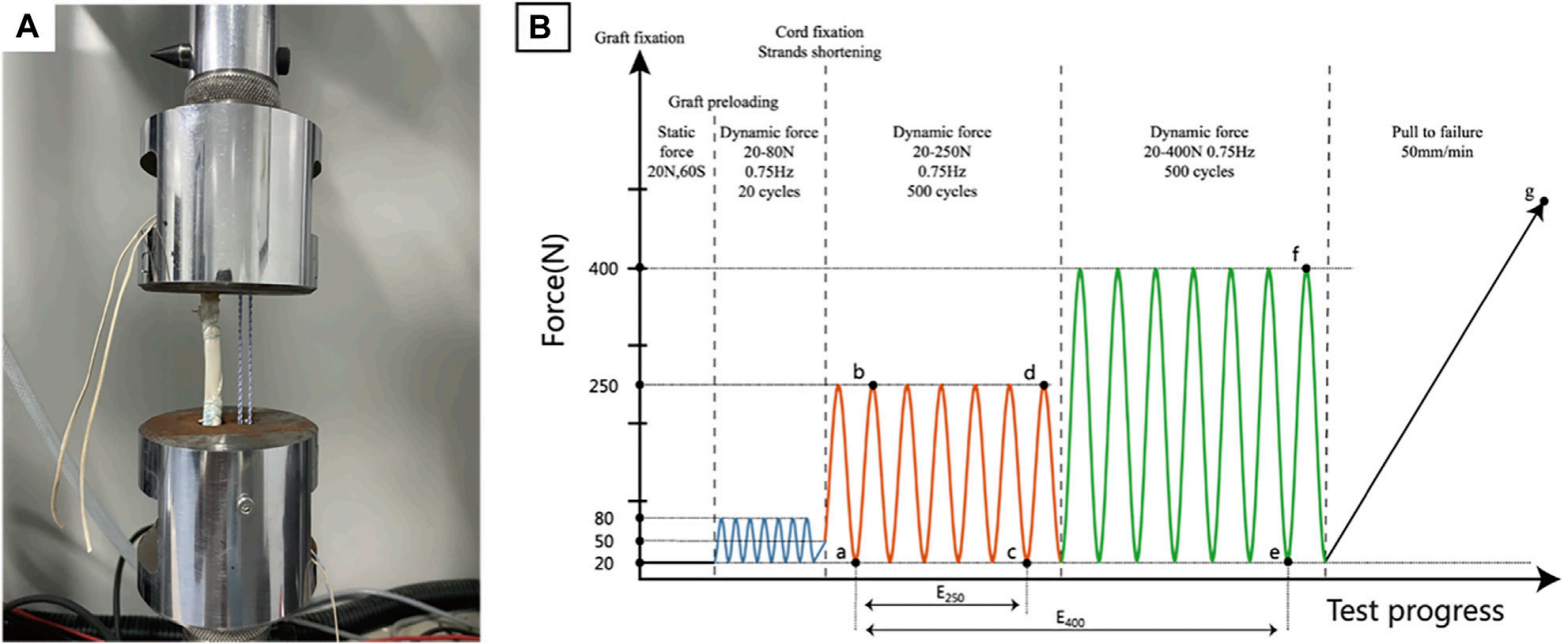
Biomechanical testing. **(A)**. Fixation of cord reinforced graft; **(B)**. Testing protocol with points of data analysis.

The custom cylindrical clamps contain two adjacent 35 mm long tunnels that can independently accommodate and secure the graft and reinforcing cords. Dynamic mechanical tests were performed on isolated cord groups, graft groups, and cord-reinforced graft groups (n = 3). The fixing process for the above construct was as follows: 1) For the isolated cord groups, the cord bypassed the suspension shaft in the tunnel exits of both clamps and tied four half-hitch knots to form a 100 mm loop; 2) For the graft group, the buttons connected at both ends of the graft were passed through both clamp tunnel exits sequentially and were then flipped. The graft was preloaded with a constant force of 20 N for 1 minute and dynamically stretched 20 times with an applied force between 20 and 80 N at 0.75 Hz (Pilia et al., 2015; Jisa et al., 2016) to simulate intraoperative operation. After completion, the inter-button distance was adjusted to 100 mm and manually pulled alternately to shorten the adjustable loops at both ends to reach a 50 N load; 3) For the cord-reinforced graft groups (Figure 2A), graft fixation followed the same procedure described above, and then the inter-button distance was adjusted to 102 mm. Cord fixation was performed first by bypassing the suspension shaft in the adjacent tunnel and tensioned to 20 N until four half-hitch knots were completed. The inter-button distance was adjusted to 100 mm to loosen the cord, which was then manually pulled alternately to shorten the adjustable loops at both ends to reach a 50 N load.

After fixation, all the above constructs underwent the same dynamic stretching procedure, and 1,000 load controlled tensile cycles were performed at 0.75 Hz. To simulate the *in vivo* loads of ACL during the early and late rehabilitation stages after ACLR (Toutoungi et al., 2000; Taylor et al., 2013), 250 N and 400 N were set as peak loads for the first 500 cycles and the last 500 cycles, respectively. The valley load level was 20 N. Finally, the construct was pulled to failure at 50 mm/min. The entire test protocol is shown in Figure 2B, and the load-displacement data were recorded with a sampling rate of 500 Hz using Wavematrix software (Instron, Boston, United States ).

#### 2.3.3 Outcome data analysis

Dynamic elongation, dynamic stiffness, and failure load data were recorded and compared. Different letters are used to mark the position in Figure 2B and Δ is the symbol of variation, specifically as follows: valley displacement is the displacement under 20 N load in each cycle, and the valley displacement difference between the initial cycle of the 250 N module and the final cycles of both the 250 N and 400 N modules is dynamic elongation (E250 -Δac, E400 -Δae), namely, the value of plastic deformation or laxity. Dynamic stiffness was calculated in the initial cycle of the 250 N module (SI -Δab) and the final cycles of both load modules (S250 -Δcd, S400 -Δef) by valley and peak values, which could objectively reflect the loading situation of the construct. The failure load was recorded during the pull-to-failure process (LF-Δg), which determined the failure load capacity of the construct. To further evaluate the functional states of the construct, a native ACL reference model was established according to methods reported in the literature (Bachmaier et al., 2018; Noonan et al., 2020), and the displacement at a load of 20 N load was defined as the zero position to facilitate comparison. The load-displacement curves of the final 250 N and 400 N module cycles of the representative sample were input into the natural ACL function model for comparison to determine whether the construct was in a proper, loose, or restricted functional state.

### 2.4 *In vitro* degradation

Degradation testing was performed according to ISO standard 13,781 ^32^. A cord sample consisted of an intermediate 20 cm woven section with a 5 cm suture section at both ends and 20 samples per group. After sterilization, the cord was immersed in 10 mL sterile PBS (pH 7.4) and sealed, maintaining the ratio of buffer volume in milliliters to sample mass in grams to within 30–40. Samples were placed on a shaker and swayed at 37 °C, PBS degradation solution was exchanged every 2 weeks, and pH was measured (n = 3).

#### 2.4.1 Scanning electron microscopy

To assess any morphological changes in the material during degradation, a 1 cm suture was cut from the end of the cord. The sample was washed thrice with sterile distilled water and dried overnight in a fume cupboard. After gold coating, three areas of the sample were randomly selected under SEM (HITACHI, Regulus 8,220, Tokyo, Japan), and images were taken at magnifications of×100, ×300, and ×1,000 (n = 1).

#### 2.4.2 Dynamic mechanical testing

At selected time points, the cord was removed from the degradation solution, vacuum dried, and then fixed to the cylindrical clamps described above and secured into a 10 cm loop. The following test procedure was the same as the biomechanical test protocol, including the recording method of dynamic stiffness and failure load (n = 3).

### 2.5 Cell compatibility

#### 2.5.1 Human ligament-derived fibroblasts culture and transfection

Primary human ligament-derived fibroblasts (hFBs) (iCell, Shanghai, China) were maintained at 37 °C in 5% CO_2_ Modified Eagle Medium (Sigma-Aldrich, St. Louis, United States ) supplemented with 10% (v/v) fetal bovine serum, 1% (v/v) L-glutamine (Gibco, Carlsbad, United States ) and 1% (v/v) penicillin/streptomycin (Gibco, Carlsbad, United States ), and the media was replaced every third day. During the compatibility experiment, it was found that Dapi could be hardly removed from the cord after staining, so the nucleus could not be clearly observed through the fluorescent signal, and the green fluorescent protein (GFP) gene was transfected into hFBs by lentivirus to make cells spontaneously fluorescent. First, P2 hFBs were implanted in a T25 flask (Corning, Corning, United States ). When the cells had reached approximately 90% confluency overnight, the original medium was replaced with 5 mL media containing 1.2 × 106 TU/mL GFP-Lentivirus (MOI 20) (Genomeditech, Shanghai, China) and 5 μg/mL Polybrene (Solarbio, Beijing, China). The flasks were cultured under standard conditions (37 °C, 5% CO_2_) for 24 h and then replaced with the standard medium. After 72 h, green fluorescence expression of GFP-hFBs was observed under fluorescent microscope (Nikon, TE 2000, Tokyo, Japan) and cultured in media containing 1 μg/mL Puromycin for further screening.

#### 2.5.2 Cell seeding

The cord was cut into 1 cm sections and placed on a 24-well plate (Corning, Corning, United States ). All cords were sterilized using 70% ethanol for 2 hours. P4-6 hFBs and GFP-hFBs were used for seeding. After counting, a high concentration cell suspension was prepared, and 30 ul (1.5 × 105) was dropped on each sample, which was then placed in a 5% CO_2_, 37 °C for 2 h to allow cells to attach. Then, the cords were transferred to a fresh 24-well plate, and the gradient number of cells (1 × 104, 2.5 × 104, 5 × 104, 7.5 × 104) were directly implanted into wells for cell attachment analysis and proliferation comparison, then 500 μL media was added to each well.

#### 2.5.3 CCK-8 assay

Cell proliferation was assessed by CCK-8 at selected time points. Cords with hFBs were transferred to a fresh 24-well plate, and media were aspirated from wells implanted with gradient numbers of cells. The wells were rinsed thrice with PBS before adding 500 μL medium containing 10% (v/v) CCK-8 reagent (Dojindo, Kyushu Island, Japan) (n = 3). After 2 h of incubation under standard conditions (37 °C, 5% CO_2_), 100 µL of CCK-8 medium samples from each well were transferred to 96-well plates (Corning, Corning, United States ) for analysis. The optical density value at 450 nm was measured using a microplate reader (Biorad, Hercules, United States ). The remaining CCK-8 media was removed and replaced with fresh standard media.

#### 2.5.4 Scanning electron microscopy

To assess cell distribution and morphology, samples were observed via SEM. Briefly, cords with hFBs were fixed in 2.5% v/v glutaraldehyde solution for 24 h at room temperature. After being rinsed thrice in PBS, the samples were dehydrated sequentially in a series of graded ethanol concentrations (40%, 50%, 70%, 90%, 100%, v/v) for 10 min each step. Samples were further dehydrated in tertiary butanol for 2 h and vacuum dried overnight. Three areas were randomly observed and photographed on each sample at magnifications of×100, ×500, and ×1,000 after gold-coating (n = 1).

#### 2.5.5 Live/dead assay

Cell viability was assessed by live/dead assay. In brief, because GFP-hFBs already have a green fluorescence signal, samples were incubated in a medium containing only 4.5 μmol/L propidium iodide (Dojindo, Kyushu Island, Japan) for 10 min to stain dead cells. After washing twice in PBS, green and red fluorescence was visualized using confocal microscopy (Nikon T2i, Tokyo, Japan), and a specific thickness layer scan was performed (n = 1). The fluorescence signal synthesis was performed using NIS-Elements Viewer software (Nikon, Tokyo, Japan).

### 2.6 Statistical analysis

The statistical analysis was performed using GraphPad Prism software version 9 (GraphPad Software, San Diego, United States ). Data in graphs were expressed as mean ± standard mean error. t-Tests and standard ANOVA with Tukey’s multiple comparisons testing were used to examine statistical differences between groups. Results were considered significant for *p* < 0.05.

## 3 Results

### 3.1 Structural and mechanical characteristics of sutures and cords

**FIGURE 3:**
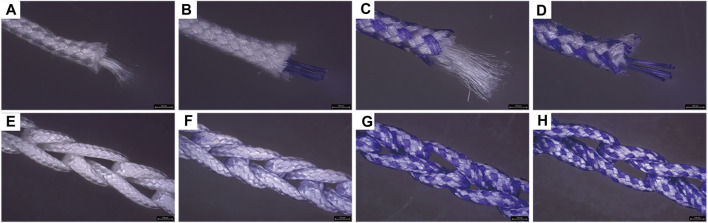
Macroscopic picture of sutures and cords. A, B, C, **(D)** Suture groups (A: UA-S, **(B)** LA-S, **(C)** MA-S, **(D)** HA-S); E, F, G, **(H)** Cord groups (E: UA-C, **(F)** LA-C, **(G)** MA-C, **(H)** HA-C), Scale bars = 750 μm.

**FIGURE 4:**
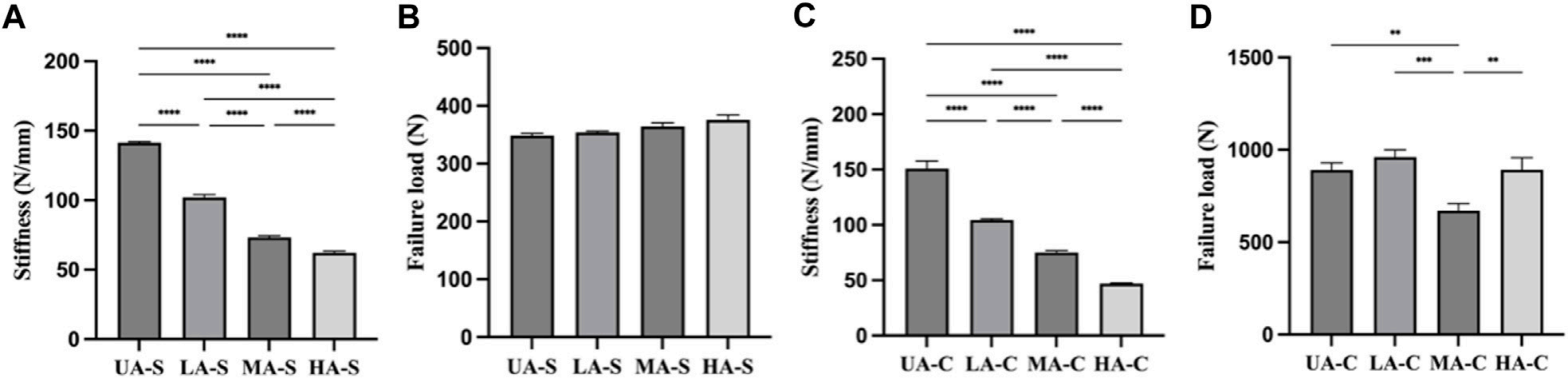
Mechanical properties of each sutures and cords: **(A)**. Stiffness of suture; **(B)**. Failure load of suture; **(C)**. Stiffness of cord; **(D)**. Failure load of cord (n = 3) (****p < 0.0001, ***p < 0.001, **p < 0.01).

Four groups of sutures were braided according to different core-sheath material configurations. UA-S was a complete UHMWPE suture (Figure 3A), and UHMWPE/PDO sutures included LA-S, MA-S, and HA-S (Figures 3B–D). Every single suture was crocheted into the corresponding cord (Figures 3E–H). UA-C served as the control group, whereas the UHMWPE/PDO cords were divided into LA-C, MA-C, and HA-C. As shown in Table 2, similar braid angle and pitch were measured in each suture and cord, respectively. Sutures and cords with UHMWPE/PDO sheath showed larger diameter and width, respectively. For sutures, the stiffness was in the 62–142 N/mm range (HA-S > LA-S > MA-S > HA-S) (Figure 4A), whereas the failure loads exceeded 348 N and showed no significant intergroup difference (Figure 4C). For cords, stiffness showed a similar gradient to that of sutures in the 47–151 N/mm range (HA-C > LA-C > MA-C > HA-C) (Figure 4B). The failure load of MA-C (671 N) was significantly lower than those of the other three groups, which exceeded 891 N (Figure 4D).

**TABLE 2:** Structural data of each suture and reinforcing cord^a^.

Suture	Diameter (mm)	Braid angle (°)	Cord	Width (mm)	Pitch (mm)
UA-S	0.73 ± 0.01bc	31 ± 0.7	UA-C	2.43 ± 0.02^bc^	2.76 ± 0.29
LA-S	0.73 ± 0.03 bc	29.9 ± 1.2	LA-C	2.45 ± 0.03^bc^	2.62 ± 0.1
MA-S	0.88 ± 0.02	32.8 ± 1.6	MA-C	2.68 ± 0.13	2.87 ± 0.16
HA-S	0.91 ± 0.02	29.7 ± 0.5	HA-C	2.78 ± 0.03	3.07 ± 0.14

^a^ Data are presented as mean ± SD.

^b^ Significantly different compared with MA-S, or MA-C (*p* < 0.05).

^c^ Significantly different compared with HA-S, or HA-C (*p* < 0.05).

### 3.2 Biomechanical testing

Test data for the graft group and each isolated cord group are shown in Table 3. The dynamic elongations (E400) of the three PDO/UHMWPE cords were significantly lower than that of the graft but higher than that of UA-C after the 400 N load module. Dynamic stiffness comprises three representative stiffness data during cyclic stretching (SI, S250, S400), representing the overall stiffness level of the structure. The dynamic stiffness of UA-C was similar to that of the graft, and those of the other three cords were lower than the dynamic stiffness of UA-C to varying degrees. The failure load (LF) of MA-C was significantly lower than that of other cords and graft groups.

**TABLE 3:** Biomechanical Behavior of cord Specimens^a^.

	Graft	HA-C	LA-C	MA-C	HA-C
E_250_ (mm)	2.49 ± 0.64^b,c,d,e^	0.55 ± 0.04	1.15 ± 0.06	1.1 ± 0.12	0.82 ± 0.26
E_400_ (mm)	5.50 ± 0.18^b,c,d,e^	1.42 ± 0.13^cde^	2.3 ± 0.22	2.62 ± 0.29	2.43 ± 0.13
S_I_(N/mm)	133.0 ± 11.1^b,c,d,e^	148.4 ± 5.9^cde^	100.5 ± 1.7^de^	70.3 ± 0.9^e^	42.9 ± 0.5
S_250_(N/mm)	154.3 ± 13.9^c,d,e^	168.7 ± 7.8^cde^	124.6 ± 5.3^de^	79.8 ± 2.9^e^	43.3 ± 1.5
S_400_(N/mm)	178.2 ± 13.1^c,d,e^	184.6 ± 6.7^cde^	141.0 ± 9.6^de^	86.6 ± 3.3^e^	52.0 ± 1.4
L_F_(N)	829 ± 99^d^	890.8 ± 38.1^d^	960.8 ± 39^d^	670.3 ± 39.2^e^	891.3 ± 65.4

^a^ Data are presented as mean ± SD.

^b^ Significantly different compared with HA-C (*p* < 0.05).

^c^ Significantly different compared with LA-C (*p* < 0.05).

^d^ Significantly different compared with MA-C (*p* < 0.05).

^e^ Significantly different compared with HA-C (*p* < 0.05).

Test data for graft with or without reinforcing cord are shown in Table 4. Compared with the graft group on dynamic elongation, the UA-C, LA-C, MA-C, and HA-C reinforced groups revealed a 69%, 39%, 19%, and 14% decrease in E250, and a 76%, 54%, 38%, and 24% decrease in E400, respectively. Compared to the graft group on dynamic stiffness, the UA-C reinforced group exhibited a 39% increase in SI and a 24% increase in S400. The increased dynamic stiffness in the PDO/UHMWPE cords reinforced groups was less than 7%. However, the UA-C reinforced group had a 17% decrease in S400. Compared to the graft group, the UA-C, LA-C, MA-C, and HA-C reinforced groups showed a 76%, 105%, 60%, and 44% increase in the L_F_ value compared to the graft group, respectively.

**TABLE 4:** Biomechanical behavior of graft and Graft + cord specimens^a^.

	Graft	Graft + HA-C	Graft + LA-C	Graft + MA-C	Graft + HA-C
E_250_ (mm)	2.49 ± 0.64^b,c^	0.77 ± 0.00^d,e^	1.52 ± 0.04	2.03 ± 0.17	2.14 ± 0.20
E_400_ (mm)	5.50 ± 0.18b^c,d,e^	1.35 ± 0.08^c,d,e^	2.52 ± 0.12^d,e^	3.42 ± 0.10^e^	4.19 ± 0.15
S_I_(N/mm)	133.0 ± 11.1^b^	184.7 ± 12.6^c,d,e^	133.2 ± 11.2	132.8 ± 5.8	134.2 ± 2.3
S_250_(N/mm)	154.3 ± 13.9^b^	205.4 ± 12.2^c,d,e^	162.3 ± 12.9	155.7 ± 10.5	152.4 ± 5.1
S_400_(N/mm)	178.2 ± 13.1^b^	221.6 ± 18.9^d,e^	190.2 ± 12.1^e^	181.4 ± 10.1	148.8 ± 7.6
L_F_(N)	829 ± 99^b,c,d,e^	1,462 ± 174	1,698 ± 87^d,e^	1,323 ± 128	1,195 ± 141

^a^ Data are presented as mean ± SD.

^b^ Significantly different compared with Graft + HA-C (*p* < 0.05).

^c^ Significantly different compared with Graft + LA-C (*p* < 0.05).

^d^ Significantly different compared with Graft + MA-C (*p* < 0.05).

^e^ Significantly different compared with Graft + HA-C (*p* < 0.05).

**FIGURE 5:**
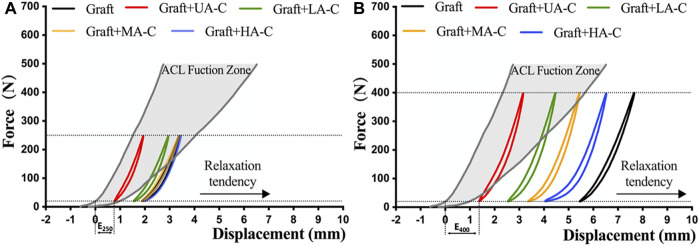
Function evaluation of construct after dynamic loading. A and B showing the load-displacement curve of construct at the end of 250 N and 400 N load block respectively, dynamic elongation (E250, E400) and the position relationship between the curve and the native ACL function zone indicate the functional situation of construct.

The last cycle curves of the 250 N load module from a representative test sample of the graft group and cord reinforced groups were within the defined native ACL function zone (Figure 5A). However, only the UA-C and LA-C reinforced groups were still within this zone after 400 N load module, while the graft group revealed a completely loose state. (Figure 5B).

### 3.3 Degradation testing

**FIGURE 6:**
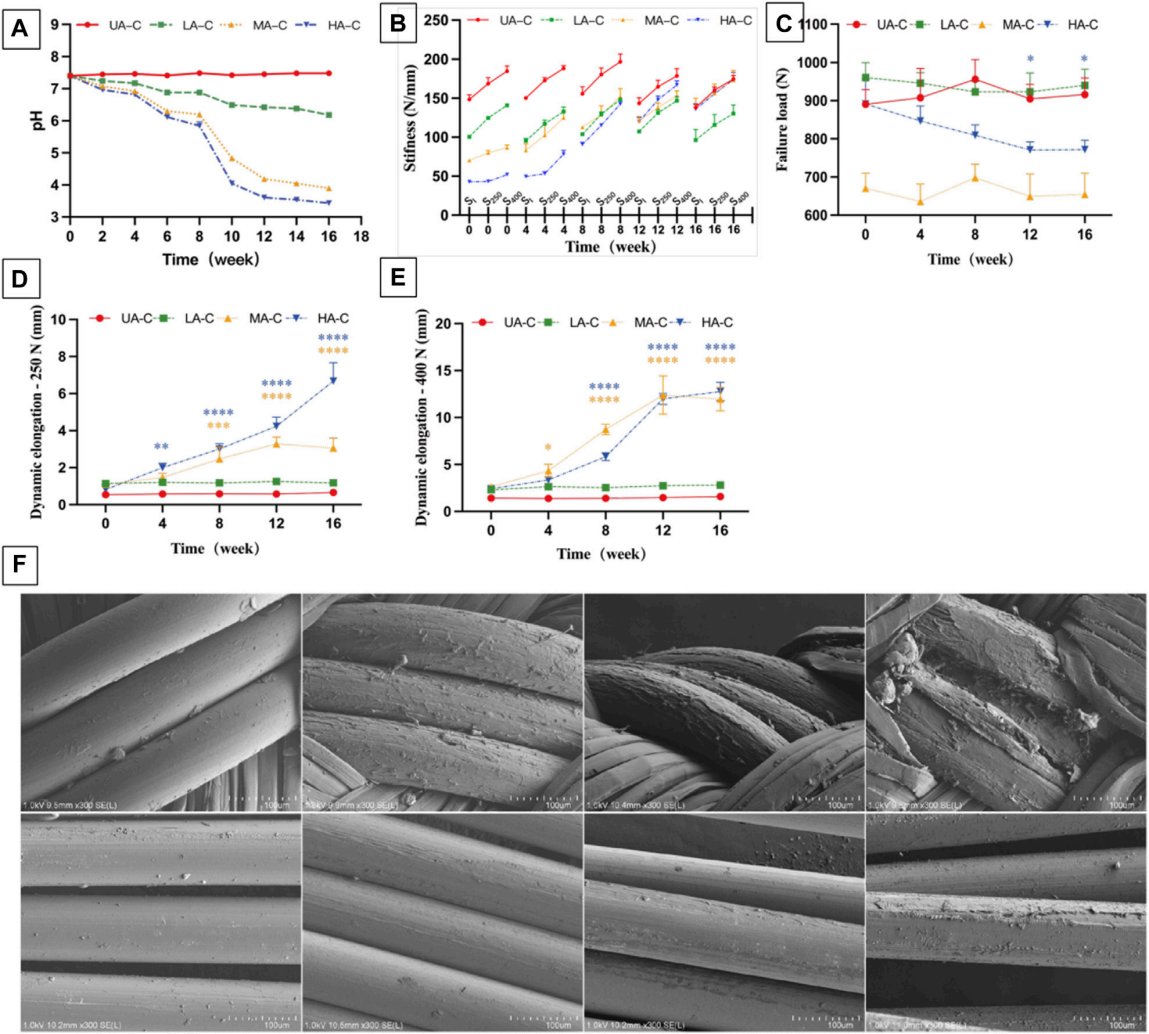
Degradation of cord over 16 weeks. **(A)**. PH value of the degradation solution; **(B)**. Dynamic stiffness of cord at different degradation points, SI: stiffness of initial cycle of 250 N load block, S250 and S400: stiffness of last cycle of 250 N and 400 N load block (n = 3); **(C)**. Failure load of cord at different degradation points; **(D)**. Dynamic elongation of cords at different degradation points after 250 N load block (E250); **(E)**. Dynamic elongation of cords at different degradation points after 400 N load block (E400); **(F)**. SEM images of HA-C at different degradation points over 4 moth, Scale bars = 100μm; t-Tests was conducted on dynamic stiffness (S400), failure load and dynamic elongation (E250, E400) between 0w and each degradation point for each group (n = 3), ****p < 0.0001, ***p < 0.001, **p < 0.01, *p < 0.05, different colors represent the corresponding groups.

PDO hydrolysate is acidic (Abhari et al., 2017). The degradation solution of three UHMWPE/PDO cords showed a decreasing PH value during the 16-week degradation period, particularly from weeks 8–10, and the degree of reduction was proportional to the ratio of the degradable material. However, the UA-C group remained essentially unchanged. (Figure 6A).

During degradation, the dynamic stiffness of MA-C and HA-C gradually increased and was almost similar to that of UA-C at the end of the experiment (Figure 6B). At week 16, the E250 of MA-C and HA-C increased to 3.06 ± 0.53 mm, and 6.68 ± 1 mm respectively (Figure 6D), and E400 increased to 11.97 ± 1.26 mm and 12.77 ± 0.98 mm (Figure 6E), while the dynamic stiffness and dynamic elongation of UA-C and LA-C remained essentially stable (Figures 6B,D,E). At the end of the experiment, the L_F_ of UA-C decreased slightly, but the other groups showed no significant changes (Figure 6C).

The degradation process of PDO monofilaments in cords is exhibited by the SEM image of HA-C (Figure 6F). The surface of the PDO monofilaments in the sheath gradually changed from smooth to rough and finally cracked at 16 weeks. However, the situation of the core PDO was better, and at 16 weeks, only slight exfoliation occurred. The degradation process of PDO monofilaments at the corresponding parts of LA-C and MA-C was essentially the same as HA-C, and the UHMWPE fiber of all cords had no noticeable change, which is not shown in the picture.

### 3.4 Cell compatibility

**FIGURE 7:**
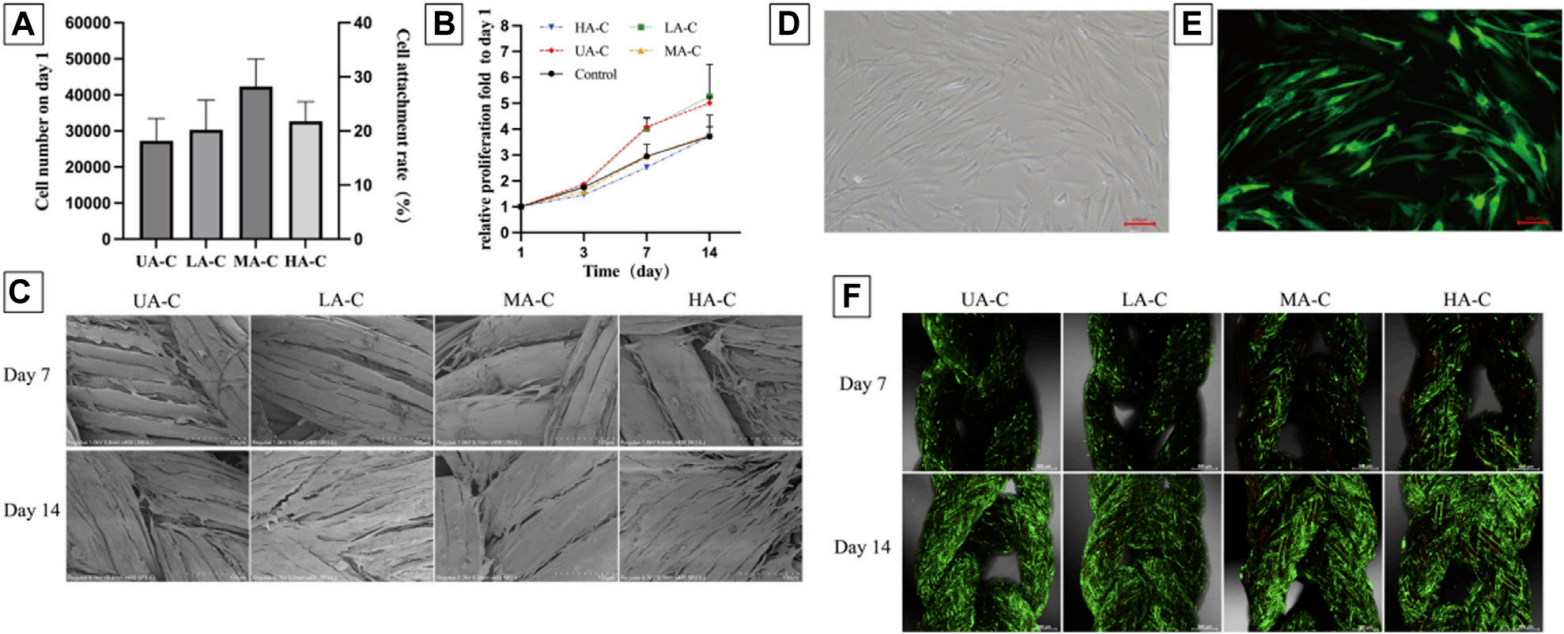
Cell attachment, proliferation and vitality on cords, **(A)**. Numbers of cells attached to the materials 24 h after seeding and cell seeding efficiency (n = 3); **(B)**. Relative cell growth on the cords over 14 days (n = 3); **(C)**. SEM images showing cell morphology and spreading on the cords at day 7 and 14 after seeding, scale bars = 100 μm; D-E. Fluorescence images of GFP-hFBs after transfection, scale bars = 100 μm; **(F)**. PI staining showed the vitality of GFP-hFBs on the cord surface at day 7 and 14 after seeding, green fluorescence representing GFP-hFBs and red fluorescence representing dead cells, scale bars = 500 μm.

The attachment rate of hFBs to the cord was about 18%–28%, with no significant difference between groups (Figure 7A). Setting the 5 × 104 hFBs implanted wells as control, and the cell proliferation fold of four cord groups showed no significant difference with the control group over 14 days (Figure 7B). On day 7, cells on the UHMWPE yarn distributed in the inter-fiber space and aligned with the fiber direction. Cells on the PDO monofilament tended to wrap around the larger fibers and bridge larger gaps (Figure 7C). The cells proliferated significantly and almost covered the surface of the cords on day 14 and aligned further (Figure 7C).

Green fluorescence of GFP-hFBs can be observed under a fluorescence microscope (Figures 7D,E). From day 7–14, the density of GFP-hFBs increased significantly, and the cells took a long fusiform shape and were densely distributed along the fiber direction on all cords. Few dead cells could be observed at both time points (Figure 7F). The results confirmed that the materials did not significantly affect cell viability.

## 4 Discussion

Independent suture tape reinforcement can effectively reduce the plastic elongation of graft constructs while enhancing ultimate failure strength (Cook et al., 2017; Bachmaier et al., 2018; Soreide et al., 2019; Smith et al., 2020a; Noonan et al., 2020; Lai et al., 2021; Matava et al., 2021; Bachmaier et al., 2022) and promote the restoration of joint stability and function (Kitchen et al., 2022; von Essen et al., 2022; Parkes et al., 2021; Bodendorfer et al., 2019). However, material debris accumulation may result in long-term foreign body inflammation (Marieswaran et al., 2018; Sinagra et al., 2018). Given the *in vivo* mechanical and structural instability of biodegradable devices (Dürselen et al., 2003; Buma et al., 2004; Dürselen et al., 2006), a partially degradable composite device could be a compromise solution to balance strength and biodegradability requirements. Both undegradable UHMWPE and biodegradable PDO are commonly used materials for manufacturing surgical sutures. Considering the excellent mechanical properties of the UHMWPE fiber and the suitable degradation period of the PDO monofilament, these two materials were selected to produce three types of core-sheath sutures with different biodegradable proportions according to the selected material configurations. The complete UHMWPE suture served as a control, and each group suture was further crocheted into a cord with the same parameters.

An increasing proportion of PDO would lead to a larger size and lower flexibility for the reinforcing cord; thus, only three available material configurations were selected, but the same manufacturing parameters still resulted in the same braid angle and pitch. Because of the large stiffness gap between the PDO monofilament and the UHMWPE fiber, the adjustment of the material configuration led to the stiffness gradient of the sutures. Stiffness was negatively correlated with the biodegradable ratio. However, sutures with a reduced content of UHMWPE yarn in the sutures can still maintained their failure load. When each suture was crocheted into the cord, the stiffness gradient and range remained unchanged. The failure load showed a more than two-fold increase, except in the case of MA-C (1.8-fold), which may be due to the cutting effect on the UHMWPE/PDO sheath by the UHMWPE core under a specific load (>600 N).

The concept of the reinforcement technique combines the reinforcing device and tendon graft to form a load-sharing system in ACLR. The goal is to reduce graft elongation, which decides the laxity degree of the knee. The mechanical properties of the 8 mm tendon graft used in this study are close to the data in the literature (Noonan et al., 2020). The dynamic stiffness of suture tape was significantly higher than that of the 8 mm graft (Bachmaier et al., 2018; Noonan et al., 2020; Bachmaier et al., 2022). In this study, the dynamic stiffness of UA-C and the graft was similar, and the UHMWPE/PDO cords had lower stiffness than the latter to varying degrees. In addition, a similar failure load and smaller dynamic elongation compared to those of the grafts indicate that the cords have a feasible safety belt function for cords.

Regarding biomechanical results, the reinforcement effects of UHMWPE/PDO cords on the graft were mainly reflected in the reduction of dynamic elongation and the increase of failure load. In addition, the dynamic stiffness of the structure was determined by both the graft and the cords. The UHMWPE/PDO reinforced group differed from the UA-C reinforced group that showed similar dynamic stiffness to the graft group in the test environment of this study, which indicated that the graft was the dominant stabilizer in this state. Finally, it is worth noting that the HA-C reinforced group showed lower stiffness at the end of cyclic stretching than the graft group, which may be due to its limited protective effect. HA-C becomes the dominant stabilizer when the graft reaches a specific elongation, and the stiffness of the construction converges on the cord.

In this study, load and displacement data of native ACLs during walking activities were used to establish a functional model to more intuitively evaluate whether the biomechanical behavior of reconstructed ACLs restores native ACL function. Compared with the 250 N load module, the cord showed a more prominent protective effect after the 400 N load module because tendency for laxity of the reinforced graft groups was significantly inhibited compared to the graft group, and the degree of significance was positively correlated with the cord stiffness. Finally, only UA-C and LA-C reinforced grafts remained in the functional area after dynamic tensile testing.

If ACLR was enhanced by a device containing degradable components, changes in mechanical properties and structure caused by material degradation should be considered. According to the pH value of the degradation solution and SEM images, a rapid degradation phase began at week 8. The UHMWPE fibers crushed PDO monofilaments hydrolyzed in the sheath during cyclic stretching, resulting in the elongation of the MA-C and HA-C. After a 250 N load module, MA-C elongation was less than HA-C. Nonetheless, this difference was not evident after applying a 400 N load module, and it appears that the UHMWPE core could limit cord elongation under a smaller load. The decreasing braiding angle and increasing weaving pitch during cord elongation led to greater dynamic stiffness. The dynamic stiffness of MA-C and HA-C gradually increased to match that of the UA-C from weeks 8–16, which was consistent with the time point of significant change in mechanical properties of different PDO devices after 9 weeks of *in vitro* degradation reported in the literature (Zilberman et al., 2005; Li et al., 2014; Loskot et al., 2022). In addition, the incorporation of a few UHMWPE yarns can also guarantee the mechanical strength of the device because even HA-C, with the highest PDO proportion, merely showed a slight decrease in the failure load at the end of the degradation period. However, the stiffer and longer cord still lost its protective effect. For ACLR, the ideal protection time is to last until graft ligamentization reaches the maturation phase, which begins 3 months after surgery in animal studies (Scheffler et al., 2008; Claes et al., 2011), and the mechanical strength of the graft will continue to increase during this phase (Goradia et al., 2000; Weiler et al., 2001; Buma et al., 2004). Research on the ligamentization process of human tendon grafts after surgery is more complex than animal experiments, which are generally thought to take longer (Falconiero et al., 1998; Sánchez et al., 2010). In this way, the mechanical properties and structural stability of UHMWPE/PDO cords are less durable except LA-C; hence, so materials with a longer degradation period may be needed to enhance the stability of the device when pursuing a higher biodegradable ratio.

*In vivo* studies have reported that UHMWPE (Smith et al., 2019; Smith et al., 2020b)and PDO (Buma et al., 2004; Dürselen et al., 2006) have good joint biocompatibility. However, given the close contact between reinforcing devices and tendon grafts undergoing continuous ligamentalization in joints, human ligament-derived fibroblasts were used for cytocompatibility testing. The low cell seeding rate was related to the hydrophobicity of the material and the gaps between fibers in the woven structures (Savić et al., 2021). Cell proliferation showed an approximately 3.7–5.3-fold increase in cell numbers over 2 weeks, while well cell vitality was observed. UHMWPE yarn with a smaller fiber diameter and inter-fiber gap showed better guidance and support for cell morphology and arrangement. It has been found in tissue engineering studies that aligned morphological features of fibers play an important role in the induction and maintenance of fibroblast morphology and phenotype (Olvera et al., 2017). Hence, the gene expression profile of cells is worth further research in this type of study.

This study demonstrated the feasibilityof a partially biodegradable reinforcing device for assisted ACLR through physical and biological assessments. A composite material scheme was adopted, and four types of cords were manufactured by a two-step process according to the planned material configuration and parameters, namely, one UHMWPE cord and three UHMWPE/PDO cords. The proportion of PDO in the cord is inversely related to its dynamic stiffness, while the latter is positively related to the reduction of graft elongation. Therefore, the stiffer cord provided better protection against elongation. During degradation, all the cords maintained their failure load. Only LA-C showed the same stable dynamic stiffness and elongation as that of UA-C, while material degradation in the sheath resulted in the other two UHMWPE/PDO cords growing longer and stiffer during dynamic stretching. Cells showed good proliferation and vitality on all cords and were aligned parallel to the fiber.

## 5 Conclusion

Overall, this study supports the potential of partially degradable UHMWPE/PDO cords, particularly LA-C, in internal bracing technology for graft protection. Nevertheless, a higher content of biodegradable material results in lower stiffness may impair the protective effect and lead to mechanical instability during early degradation.

## Data Availability

The original contributions presented in the study are included in the article/supplementary material, further inquiries can be directed to the corresponding author.
